# How can electronic health records serve as a tool for clinical trials?

**DOI:** 10.1192/j.eurpsy.2024.135

**Published:** 2024-08-27

**Authors:** R. Stewart

**Affiliations:** ^1^Department of Psychological Medicine, King’s College London; ^2^PMOA, South London and Maudsley NHS Foundation Trust, London, United Kingdom

## Abstract

**Abstract:**

Increasing volumes of information are being collected via electronic health records and there is growing multi-site expertise in utlising these for research. This emerging field of healthcare data science is not only concerned with the technical challenges associated with complex data, but also with the need for effective security and governance in the use of sensitive information with robust structures for stakeholder input and guidance. To date, most of the focus has been on supporting observational cohort studies nested within clinical records data - particularly investigating research questions around treatment response and course/prognosis. It is likely that electronic health records will become increasingly integrated with clinical trials, providing opportunities for pre-study feasibility scoping, targeted recruitment, and enhanced and extended follow-up. In addition, there is interest in emulated trials using routine data. For mental health data science, key challenges lie in the quality and quantity of data made accessible, with a particular need for natural language processing to derive structured data from extensive clinical text. Many of the challenges have been addressed for observational research, creating exciting prospects for a transformed trials landscape.

**Disclosure of Interest:**

R. Stewart Grant / Research support from: Janssen, GSK, Takeda

